# The Use of Smartphone Photogrammetry to Digitise Transtibial Sockets: Optimisation of Method and Quantitative Evaluation of Suitability

**DOI:** 10.3390/s21248405

**Published:** 2021-12-16

**Authors:** Sean Cullen, Ruth Mackay, Amir Mohagheghi, Xinli Du

**Affiliations:** 1 Department of Mechanical and Aerospace Engineering, College of Engineering Design and Physical Sciences, Brunel University, Kingston Lane, Uxbridge UB8 3PH, UK; ruth.mackay@brunel.ac.uk (R.M.); xinli.du@brunel.ac.uk (X.D.); 2 Sport, Health & Exercise Sciences, College of Health, Medicine and Life Sciences, Brunel University, Kingston Lane, Uxbridge UB8 3PH, UK; amir.mohagheghi@brunel.ac.uk

**Keywords:** lower limb prosthesis, socket, CAD-CAM, photogrammetry, genetic algorithms, scanning, digital twin, measurement techniques

## Abstract

The fit of a lower limb prosthetic socket is critical for user comfort and the quality of life of lower limb amputees. Sockets are conventionally produced using hand-crafted patient-based casting techniques. Modern digital techniques offer a host of advantages to the process and ultimately lead to improving the lives of amputees. However, commercially available scanning equipment required is often expensive and proprietary. Smartphone photogrammetry could offer a low cost alternative, but there is no widely accepted imaging technique for prosthetic socket digitisation. Therefore, this paper aims to determine an optimal imaging technique for whole socket photogrammetry and evaluate the resultant scan measurement accuracy. A 3D printed transtibial socket was produced to create digital and physical twins, as reference models. The printed socket was photographed from 360 positions and simplified genetic algorithms were used to design a series of experiments, whereby a collection of photos were processed using Autodesk ReCap. The most fit technique was used to assess accuracy. The accuracy of the socket wall volume, surface area and height were 61.63%, 99.61% and 99.90%, respectively, when compared to the digital reference model. The scanned model had a wall thickness ranging from 2.075 mm at the top to 7.758 mm towards the base of the socket, compared to a consistent thickness of 2.025 mm in the control model. The technique selected did not show sufficient accuracy for clinical application due to the degradation of accuracy nearer to the base of the socket interior. However, using an internal wall thickness estimation, scans may be of sufficient accuracy for clinical use; assuming a uniform wall thickness.

## 1. Introduction

Obtaining a reliable and comfortable socket fit remains one of the largest areas of dissatisfaction amongst lower limb amputees [1,2,3]. Traditionally, transtibial sockets are produced from handmade plaster casts of an individual’s residual limbs, these casts are then manipulated to produce a rectified copy of the original patient’s limb. The rectified cast is subsequently used as a mould to form the socket material [4,5,6,7]. When the socket fit changes the socket is discarded and a new socket is produced. This is often a lengthy and wasteful process, especially if only minor revisions are needed. Digital technologies can replace stages in the traditional socket manufacturing process, such as rectification, mould production, and final socket fabrication. Digital techniques offer a host of advantages over conventional techniques, such as reproducibility, design history, and rapid prototyping. The ability to digitise an existing socket without destroying it could in turn create sockets that are more comfortable for the end user, leading to an increased quality of life, whilst reducing pain and suffering.

Within limb centres one of the critical advantages of digital socket production is the ability to utilise centralised fabrication facilities, avoiding much of the advanced machining costs whilst reducing workshop man hours, and potentially decreasing delivery times to patients [8,9,10,11,12]. There are techniques that use direct limb casting to laminate the socket onto the user, allowing for socket delivery within one session [13]. Whilst these methods have increased satisfaction for transfemoral amputees, the authors are not aware of any literature on reliably successful use with transtibial amputees.

To fabricate prosthetics using computer aided manufacturing (CAM) techniques, the patient’s biological data require digitising. A popular method for digitising is the use of hand held 3D scanners that use lasers or structured light to determine the surface topography and volume of a patient’s residual limb, residuum cast, or existing socket [14,15]. The scanner is moved by the clinician around the subject to capture the entire surface. The topography point cloud generated by the scanner is then converted into a mesh, and finally a 3D object that can be manipulated via computer aided design (CAD) software to create the desired object, such as a socket mould. One of the main barriers to accessing digital techniques is the equipment cost. Commercially available prosthetic 3D scanners, such as the WillowWood Omega scanner, are often proprietary and can cost in excess of £5,000. This is significant when compared to the cost of casting consumables, in the region of <£50; re-training and upskilling in the use of 3D scanners should also be taken into consideration [16,17]. Therefore, the use of low-cost scanning equipment could be the key to more widespread use of digital socket manufacturing, especially in low- and middle-income countries (LMICs) where traditional fabrication equipment is not established. Van der Stelt et al., who used a 3D scanner and 3D printing to produce transtibial prosthetics, required €15,000 alongside other significant donations, including the time of a researcher, that were required to produce 3D printed sockets in Sierra Leone [18].

Photogrammetry is a digitisation technique in which a collection of 2D photographs from different viewpoints of an object are analysed with specialised software. Key elements are identified that can be seen in multiple images, allowing points to be located in a 3D space relative to each other, forming a 3D surface mesh [19,20,21].

Solav et al., explored using photogrammetric techniques for residuum scanning applications with an array of cameras centred around an enclosed scanning volume [22]. This method cost $2,000, which was low when compared to 3D scanners; however, it was specialised which would result in increased cost if commercialised, affecting accessibility. An alternative solution to specialised equipment for digital scanning was explored by Hernandez and Lemaire. Their work utilised a readily available smartphone camera and patterned tape to digitise prosthetic sockets [23]. Modern smartphones can cost up to £1,000, but are something that most prosthetists will have access to.

Another important factor to consider for scanning techniques is accuracy. It was noted by Sanders and Fatone, during a literature review, that a general residuum volume change of −5% or +2.5% could lead to a clinically significant change in fit, with volume changes of −10% to +5% leading to an unacceptable socket fit [24]. These volumes were taken from changes in sock ply and converted to an equivalent volume percentage. Therefore, inaccurate residuum scans can lead to a socket with poorer fit. Hernandes and Lemaire compared the scan volumes and surface measurements for the internal socket wall. The percentage volume difference in the study was reported between 2% and 10% [23]. By comparison, a low cost (~£500) structured light 3D scanner (iSense) was shown to have errors of 3–6% in a study by Armitage et al. [25]. Laser scanners can achieve even higher accuracies, with one study reporting accuracies of 0.8% in volume [26]. Whilst the smartphone photogrammetry errors are higher, the study by Hernandes and Lemaire imaged an internal socket wall which has a limited number of viewing angles, and is therefore more challenging to scan when compared to the other studies [23]. A team in Diponegoro that used photogrammetry techniques, with both smartphones and conventional DSLR cameras, to produce a transtibial socket, was unable to fairly evaluate the scan accuracy for the socket as the rectifications were different for the hand-casted model. The team were able to determine that the photogrammetry scans had an accuracy of over 95% against uniform reference objects [27].

The usability of each type of scanning method should also be considered alongside the cost. Structured light scanners are handheld and portable, and provide real-time scanning, whereas laser scanners can be static with a set measurement volume, such as the one in the study mentioned previously [26]. Davide et al. investigated the difference between high- and low-cost scanning equipment; this highlighted that whilst high and low cost fixed scanners may have increased accuracy, the set up and scan time would limit their applications in prosthetics when compared to hand held devices for direct limb scanning [28]. Smartphone photogrammetry can be considered handheld as it uses a mobile device with no fixed measurement volume; currently, however, there are no real-time scanning options available on common smartphones suitable for prosthetic applications.

Determining the topological differences that cause volume change are critical for clinical use in socket reproduction, as certain residuum regions are more sensitive to increased pressures created by the socket wall topography [29]. It should be noted that many of the studies discussed used residuum casts or substitutes for evaluation and that additional errors will be present when using the techniques on patient’s residuums. One literature review concluded that there are limited papers on using digital scanning techniques on amputees, making it impossible to determine if digital techniques are reliable and valid when compared to residuum volume calculations using hand- or water-based measurement techniques [30]. However, another paper reports that high end 3D scanners (Omega, WillowWood, Mt. Sterling, OH, USA) can be more reliable and accurate than traditional techniques [31].

One of the main challenges of using photogrammetry is the refinement of the photographing technique for accuracy and reliability. Whilst the general principal is to ensure a 60% overlap between adjoining images, there are near infinite possibilities to the number of photos taken and their relative position to the subject [32]. In other engineering disciplines, problems with multiple variables, such as this, have utilised genetic algorithms to reduce the computational requirements, reducing the time taken to find an optimum solution [33,34]. For example, genetic algorithms treat the experiments akin to evolving organisms, breeding and mutating gene information to find an experiment with the highest fitness characteristics (accuracy) based on a predetermined fitness equation iteratively. Unlike systematic experiment design methods, genetic algorithms search a wide array of possible experiments quickly via random sampling; these are refined to find the optimal solution. This method has limitations; not all possible experiments can be trialled due to the infinite number of inputs that could be used, therefore, there is no guarantee that the final experiment is the global optimal, however it does achieve a local optimum with significantly reduced computational time [35].

This paper aimed to determine an optimal arrangement of imaging (photograph) positions for a transtibial socket to give the highest level of photogrammetry scan accuracy for an entire socket. This was conducted by prioritising reduced photo count, using simplified genetic algorithms, and quantitively evaluating the accuracy of the resultant scans. This aim was with considerations for using low cost and widely available technologies, such as a smartphone camera, as well as falling within the limits of using Autodesk ReCap Photo software (Autodesk, San Rafael, CA, USA), to help overcome some of the challenges facing digital integration in prosthetic limb centres.

## 2. Materials and Methods

### 2.1. Control Model Creation and Preparation

It should be noted that these experiments were conducted during the COVID-19 pandemic when access to laboratories was prohibited.

An existing transtibial socket was marked with pattered tape (unicorn-patterned duct tape, Shurtape, Hemel Hempstead, Hertfordshire, UK) allowing for easier identification of overlap within images by the photogrammetry software. Whilst the pattern was repeating, slight inconsistencies in application on the socket as well as background objects were sufficient for point mapping.

**Figure 1:**
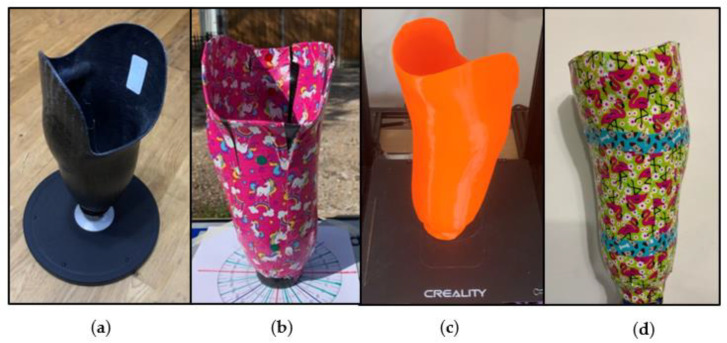
Socket and model preparation stages: (**a**) socket; (**b**) taped socket; (**c**) 3D printed control socket; (**d**) taped 3D printed control socket.

This approach is similar to one outlined in the literature, including the marker dots for scaling, see Figure 1a,b [23]. Initial tests to find suitable ranges for camera positioning were conducted, resulting in a scan that resembled the socket surface both internally and externally. This was achieved by photographing the socket 384 times using a preliminary version of the technique described in more detail using cylindrical co-ordinates (Section 2.2). The photos were manually selected on a trial-and-error basis to achieve a scan that resembled the socket. After six attempts, a scan using 96 images taken at 30 degree increments at the following heights (h) relative of the base of the socket and radial distances (d) was chosen: h = 10 cm at r = 50 cm; h = 20 cm at r = 100, 50, and 30 cm; h = 50 cm at r = 100, 50, 30, and 10 cm. The achieved scan contained deformations, so the internal socket wall was removed from the digital model. The wall was replaced with a uniform offset of 2.6 mm from the external surface geometry, to create an approximated digital socket model. This model was then 3D printed (Creality Cr-10, Longhua Dist, Shenzhen, China) using PLA (Polylactic Acid) with a 0.4 mm nozzle at 0.2 mm layer height to create a near identical physical and digital model (digital twin), which is referred to as the control socket, Figure 1c. The digital and physical control socket had a known volume and surface area, that could be used for comparison with scanned files, based on the object file parameters.

The 3D printed control socket was then marked with patterned tape (flamingo-patterned duct tape, Shurtape, Hemel Hempstead, Hertfordshire, UK), with an additional two bands of patterning on the external socket wall (dog-patterned duct tape, Shurtape, Hemel Hempstead, Hertfordshire, UK), see Figure 1d. As the control model was significantly lighter than the original carbon fibre socket, a 300 g adaptor was added to avoid movements in the model position whilst the photographs were acquired. To achieve full socket coverage, the taping process took approximately 10 min. The representative steps are shown in Figure 1.

### 2.2. Equipment and Structure for Photo Capture

**Figure 2:**
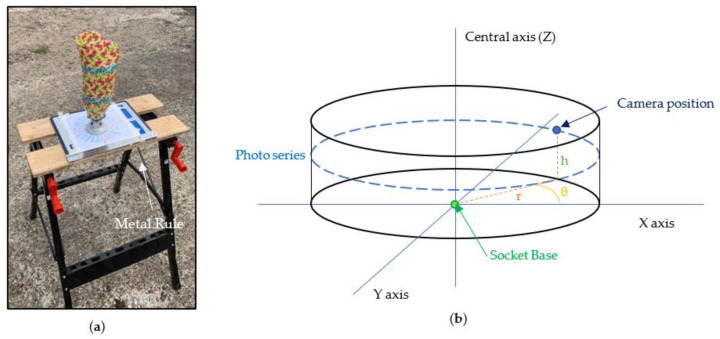
Illustration of experiment set up; (**a**) taped socket positioned on platform with ruler; (**b**) cylindrical co-ordinate system used for camera positioning.

In order to photograph the control model, the socket was placed on top of a flat plate on a raised platform in the centre of a printed protractor indicating the angle increments. The smartphone (iPhone XR, Apple, Cupertino, CA, USA) was attached to a tripod with the flash turned off, in a portrait position using automatic focusing. All settings were automatic and the standard photo mode was used, to represent how this technique is likely to be carried out by clinicians. To reduce time between photos, a remote Bluetooth trigger was used. A sample photo taken from the experiment shows the model arraignment in Figure 2a.

The positions for the camera were defined using a cylindrical coordinate system, with the base of the socket as the origin. The photos were split into a series of set heights (h; −10, 20, 50, and 80 cm) and radial distances (r; 0, 10, 30, 50, 100, and 150 cm), with each series consisting of 24 photos taken at 15° increments to allow for analysis of four angle increments (Ө = 15, 30, 45, and 60°); the 3D representation is shown in Figure 2b. These parameters were chosen based on the initial testing. Increasing the number of discrete options for r, h, and Ө would make it difficult to achieve an accurate measurement and would lead to a large increase in the total number of photos required. Some photo series were not feasible for low r values, as the camera was too close to view the entire socket at low heights. Larger r and h values were omitted as they provided no increased accuracy benefits during initial testing.

The tripod was used to fix the camera at the designated height, and the radial distance was checked every second photo with the use of a measuring ruler marked with each key distance. The angle was visually identified using the camera screen and the pre-set marks on the protractor. The expected error in positioning for each photo is ±5° angle, ±5 cm in radial distance, and ±2.5 cm in height. In total, 360 photos were taken across an approximately one-hour timespan. As photography was conducted outside, the ambient light levels were recorded at the start of each series, with indirect sunlight being preferred in order to avoid shadows and lens flaring. The ideal light level was 21–25 Klux, corresponding to overcast and evenly diffused lighting, and was measured between each photo series. If the measured levels were outside this range, photography was halted until light levels returned. At light levels outside this range the internal surface of the socket was not as visible or contained shadows. These inconsistencies would affect scan quality and be of detriment to the optimisation process.

### 2.3. Genetic Algorithm Definition

The design of the experiments used for this optimisation was simplified genetic algorithms constructed with a total of 30 gene bits with two bits per photo series. The first bit designated by “A” refers to the angle of photos for that series (15, 30, 45, 60), and the second bit “G” determines if the photo series is included or not (1, 0). In the example shown in Table 1, the gene series generated created an experiment consisting of 76 photos across the 1st, 2nd, 4th, 8th, 9th, 14th, and 15th photo series at angles of 45°, 15°, 30°, 60°, 60°, 30°, and 45°, respectively, with all other photos being excluded.

The chance of a specific angle being chosen for a gene was equal at 25% across the four discrete angle options, selected randomly for each bit pair. There was a 40% chance that any pair would be active (1) as opposed to excluded (0). The increased weighting of inactive genes was used as a fair way to reduce the photo count of a randomised gene set. As Autodesk ReCap Photo had a limit of 100 photos, any gene sets above this were regenerated. Before any gene set was included it was checked against the existing gene sets that had been tested to ensure there were no duplicates. During initial testing Autodesk ReCap and Meshrooms were trialled, ReCap was chosen as the output scans were more consistent and allowed for simultaneous solving, reducing the overall solver time for the optimisation generations. For this project, access to Autodesk ReCap was free under an educational licence.

For this method of optimisation, both gene mutation and breeding were used. Gene breeding consisted of crossing the last five bit pairs of the two most fit experiments from a given generation. The eleventh bit pair was chosen as the crossover point, as it denoted the photos of the control model that focused on the internal socket surface. An example of two bred genes is shown in Table 2.

For the mutation of genes, there was a 10% chance that any given bit could change value by one increment, i.e., inactive pairs could become active and active pairs could become inactive. If the angle bit changed, there was an even chance that the increment would be positive or negative unless this was at the end of the discrete set, i.e., angles of 45° could become 30° or 60°, but an angle of 60° could only become 45° through mutation. Table 3 shows a mutated gene example with the orange cells indicating mutations. Some mutations led to no change in the experiment, such as the angle change on the inactive gene 2 set.

For this study, five generations were used, each consisting of 15 gene sets. The first generation consisted of 15 randomly generated gene sets. Each subsequent generation consisted of the two most fit gene sets from the previous generation; the two gene sets created from breeding; six mutated gene sets (three from each carried forward); and five additional randomly generated gene sets. After the five generations, the most fit gene series was identified. Each gene pair was reduced by two further increments and retested. This allowed the minimum number of photographs required to be determined without compromising accuracy. To rank each gene series from a generation, Equation (1) was used to compare each gene set to one another:

Rank = AVG(Rank(Volume), Rank(Surface Area), Rank(Number of Photos), Rank (Model Height))(1)

The metric scores for volume, surface area, and height were calculated by determining the percentage difference between the scan data and the digital control model. If two gene sets received the same calculated rank, the one with the closest volume to the control model was selected as the most fit gene within the series.

### 2.4. Model Preparation for Analysis

Following a successful scan, the socket models were isolated by removing the surrounding mesh with the slicing tool, thus leaving only the socket and flat plate and removing any residual data. The model was then scaled using a point-to-point distance, based on the longest clear mm markings of the metal ruler (commonly 300 mm). Using two points on the ruler furthest apart reduced the effect of errors in point selection. Some scan results had poorly meshed photos, specifically of the ruler where the beginning or end of the ruler was obscured. A final cut is made parallel to the plate to the base of the socket, to remove the model data for the pyramid adaptor. The built-in ReCap Photo mesh report was used to determine the model volume, surface area and height for comparison with the equivalent properties of the control socket file.

**Figure 3:**
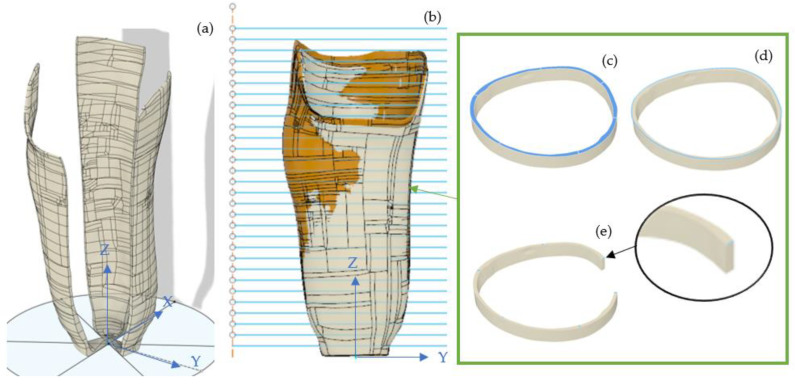
A digitised and imported socket in Autodesk Fusion 360: (**a**) socket scan 22FBG7-1 segmented around central axis, showing alternate segments; (**b**) vertical cut lines on with both sockets aligned and visible with the white model being the scan and orange being the control model; (**c**) sample slice with top surface highlighted; (**d**) sample slice with loop length highlighted; (**e**) sample slice with perpendicular wall thickness of single segment.

A selected model mesh could then be exported from ReCap as an object file for further analysis in Autodesk Fusion 360, using a 10k Quad based model; this was found to be the most reliable file type for export and import between the software used. Once imported the scan model was aligned in the digital space with the control model, using socket reference points (base of socket, patella tendon bar, trim line, fibial head relief area). A series of cuts were made at 10 mm increments concurrently on both models; the cuts were made parallel to the base of the control model, and at 60° sections about the center axis of the socket, as illustrated in Figure 3. The volume, top surface area, and external edge length of each vertical slice was extracted for both the control and scanned model for direct comparison. The internal edge length of each slice at the 60° angle was also extracted to indicate wall thickness. This data extraction provides information on both the external topography and wall thickness of the socket files.

## 3. Results

**Figure 4:**
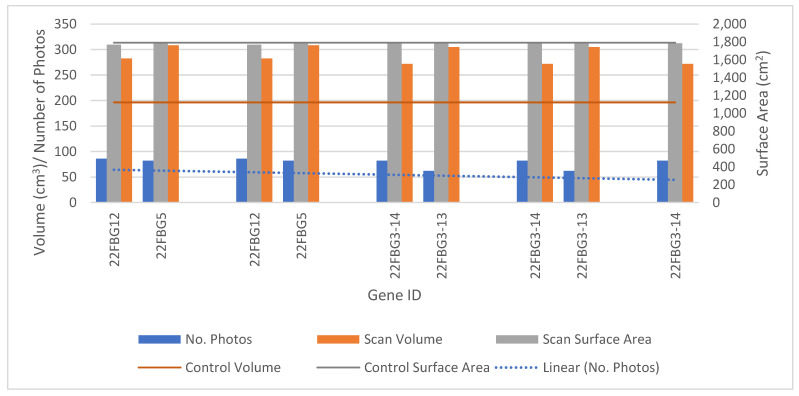
Comparison of the two most fit gene sets from each generations; volume and surface area to control values, including linear approximation of trend in number of photos.

The final selected experiment photo set referred to as “22FBG3-14” was identified during the third generation and achieved the highest fitness score throughout the remaining generations. Gene set 22FBG3-14 was comprised of 82 photos and had an accuracy in terms of socket wall volume, surface area and height of 61.63%, 99.61% and 99.90%, respectively. The volume bound by the internal socket wall was 6.76% smaller than that of the control model. Therefore, the accuracy of reproduction rectified moulds from the scan would be 93.24%. The model properties of the two most fit gene sets in each series are shown in Figure 4, showing a slight reduction in photo count through the generations. The measured volume and surface area for the control model are 196.4 cm^3^ and 1790.9 cm^2^ respectively, based on the digital mesh.

In total, 75 unique simulations were run, resulting in 32 successful socket meshes. The remaining models were rejected due to incomplete models being formed. Incomplete models showed errors, including filled internal volume, large or numerous holes in the socket wall or surface fragmentation and anomalies. Across all successful experiments the average accuracies measured against the control model were 36.13%, 99.17%, and 99.50% for volume, surface area, and height, respectively, with an average photo count of 81.

The camera position cylindrical coordinates for the final gene are shown in Table 4:

### Analysis

**Figure 5:**
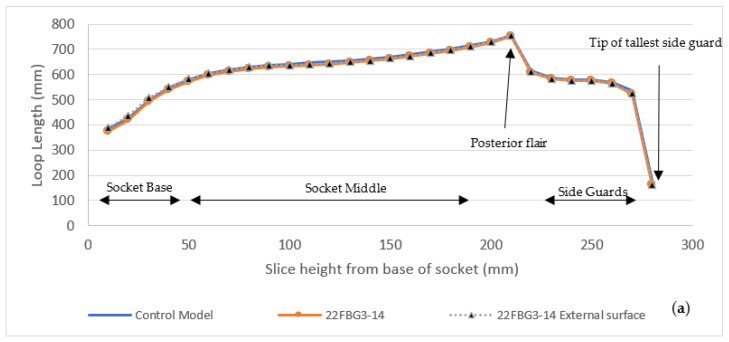

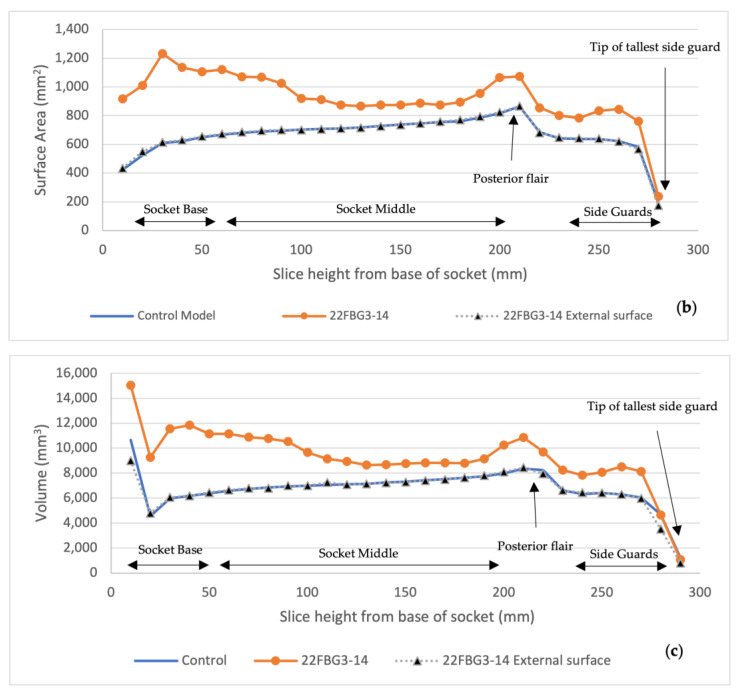
Analysis of scan model and reconstructed model vertical slices, compared to digital control model: (**a**) comparison of slice loop length based on external top edge; (**b**) comparison based on surface area of the top cut surface for each slice; (**c**) comparison of entire slice volume for each vertical height from socket base.

In order to understand the large difference in volume but minimal difference in surface area, as previously discussed, the digital scan was exported as a 10k quad based object file into Fusion 360. No additional surface smoothing was used. The digital scan file had some slight pitting and mesh defects; these were rectified using star point surface fills. Both the digital control model and scan model were split into 29 horizontal slices, each slice being 10 mm thick. Each section was then further divided into 60° sections around the central vertical axis, as per the methodology. An additional model was created by replacing the internal socket wall from the scan mesh with a uniform offset of 2.2 mm from the external wall. From these models the following data could be extrapolated: slice volume, slice top surface area, and slice top surface loop length (face outer contour length), as shown in Figure 5.

**Figure 6:**
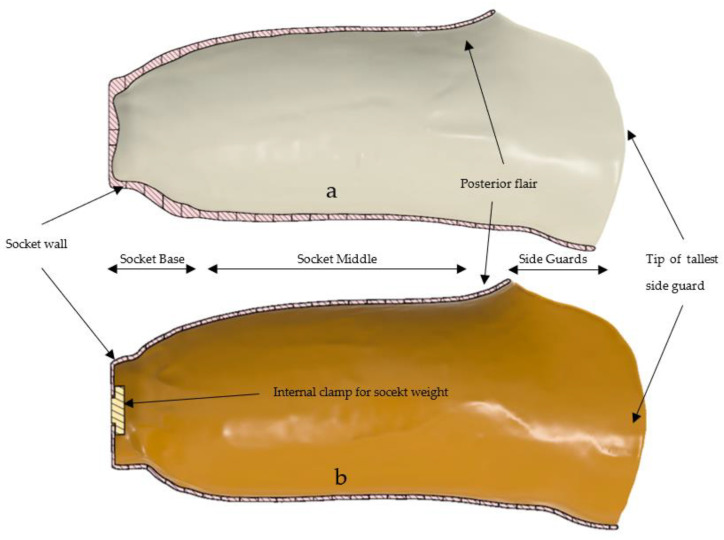
Cross sectional analysis of the socket, including the visual reference points: (**a**) cross sectional view of the digital socket scan; (**b**) the digital reference model that was 3D printed.

Each of the graphs follows the expected pattern of a quick increase in volume, surface area, and loop length in the first 50 mm at the base of the socket, followed by a steady increase through the main portion of the socket as the circumference remains similar. There is a quicker increase followed by sudden drop in all metrics at the ~200 mm point, which is representative of the posterior flair (above the popliteal depression), followed by the side guards (lateral and medial supracondylar sections). Following this, there is a decrease in metrics as the sideguard reduces in size, followed by a sharp decline at the tip of lateral sideguard. A visual depiction using the digital model cross sections can be seen in Figure 6.

**Figure 7:**
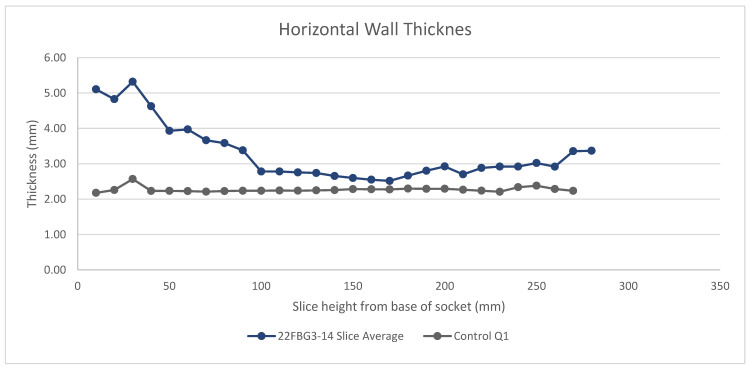
The horizontal wall thickness of the scanned model averaged across six radial cuts for each vertical slice compared to the control model showing a large disparity between the scan and control at the base of the socket.

From the six sections of each slice the horizontal socket wall thickness was extracted. The average thickness for each slice is shown in Figure 7. Similar to the trend seen in regard to the slice volume, the average wall thickness at the base of the socket is significantly higher in the scanned mesh. The scan wall thickness also increases nearer the brim of the socket, reducing the accuracy in this region.

The addition of tape to the 3D printed socket caused a slight increase in wall thickness to 2.657 mm +0.42–0.53, averaged across 10 points at the brim of the socket compared to 2.235mm from the control digital model. The increase depended on the number of layers of tape, as there were some areas of overlap. Whilst this addition may account for some of the scan increases in wall thickness, the amount is minimal compared to the range in the measured wall thicknesses (2.075 mm to 7.758 mm).

## 4. Discussion

The aim of this study was to identify an optimised photogrammetry technique for accuracy, whilst reducing the number of required photos. Overall, the optimisation process led to a clear most-fit gene set (22FBG3-14). Further reductions in photo count from this gene set led to significantly larger inaccuracies in volume, suggesting that the gene was a local optimum. With the large number of potential gene sets, it is difficult to say if the optimal solution discovered is the global or a local optimum. Determination of the actual global optimal gene set would require significantly more computational time.

The fitness equation used did achieve the target of reducing the number of photos; this may have put more weighting on the surface area of the models than needed, as the lowest recorded accuracy was 97.30% with an average of 99.16%, leading to a very small range. Instead, the use of an exclusion criteria for experiments with and accuracy below 98.50% may have allowed for the selection of a lower number of photographs, or more accuracy in terms of the volume of the gene sets. By contrast the highest accuracy in volume for the socket was 67.69% and a photo count of 100, compared to 22FBG3-14, which had a percentage volume difference of 61.63% and a photo count of 82.

The number of rejected experiments—32 out of 75—and large percentage volume differences from the photogrammetry scans suggest that the method is not reliable for transtibial socket applications; this is in contradiction to previously published literature [23]. A socket resembles a tall cylinder, which restricts the number of viewing angles where the base is still visible on the internal wall. The small changes in the viewing angle led to minimal point to point changes, making depth perception for the software difficult. In addition, light levels at the base of the socket were visibly lower than the socket exterior, whilst additional light may benefit photogrammetry, an increase in reflections was found to reduce scan quality during initial experimentation. This could be improved with brighter diffused lighting; however this was unavailable at the time due to COVID-19 restrictions. Reliability may also be improved by scanning a mould of the internal surface of the socket, either through an alginate copy of the socket or using the Plaster of Paris (PoP) mould prior to socket lamination. Whilst not in the scope of this paper, further study should be conducted into the accuracies and repeatability of scanning positive socket moulds.

A common observation across all of the gene sets was the significantly larger scan volumes compared to the control model. Previously, this issue was determined to be a scaling issue [23]. When combined with the surface area and height metrics, scaling would lead to additional distortions. Instead, the comparison of the scan wall thickness showed that the wall accuracy deteriorated closer to the base of the socket. It is reasonable to assume that the internal socket wall degradation is the main source of the volume discrepancy, as the external loop length of each slice is near identical to the control model (2.64% average difference). The ruler-based scaling method could be considered more accurate than the measured distance between marker dots used in the literature, as it negated the need to measure the distance between reference points. It should be noted, however, that having multiple reference objects for scanning provides redundancy if the ruler is obscured, as was noted on some scanning experiments.

The results achieved in this paper are in line with the existing literature when considering the volume bound by the socket walls, which is the equivalent of the rectified residuum cast. The final model had an internal volume that was 6.76% smaller than the control model, which would be clinically noticeable to the wearer of a recreated socket. The non-uniform reduction in volume is critical for socket reproduction, as the volume reduction is heavily distributed towards the base of the socket. By contrast, when using an extrapolated internal socket wall from the external surface, the internal volume difference lowered to 3.2% smaller. Critically, however, this decrease in volume was uniformly distributed through the socket; thus a better fitting socket may be created if CAM manufacturing is used. This method may not be as effective if there is additional material lamination added during the socket construction, as the distal end of the scan would become enlarged. However this could be remedied by adding material into the base of any reproduced sockets.

The digital twin technique for scan accuracy evaluation used in this paper assumed that the physical 3D model and digital model were identical. Papers from different disciplines have used a meteorological grade 3D scanner to determine a reference topography that scans from other devices that can be compared with [36,37,38,39]. Both methodologies have merit, and inherent error in either production or digitisation. The meteorological scanners were prohibitively expensive and do not fit with the low-cost principals of the study. Further study may be beneficial to determine the true error inherent in the digital twin technique and form a ‘gold standard’ for model evaluation for prosthetic applications. These issues are inherent to photogrammetry, leading to reproduced sockets that would have a significantly restricted distal end to any sockets produced. This could result in uncomfortable reconstructions that will ultimately affect the clinical applicability. However, the technique is non-destructive, unlike traditional PoP copying techniques. The level of distortion observed could be comparable to distortions found in other non-destructive copying methods, such as alginate, and further study should be used to make further comparisons.

Photogrammetry may still be of clinical benefit for sockets with near uniform wall thickness as the external surface scan can provide a reasonable approximation for the entire socket, however this will come with increased computation cost and skill requirements, due to the post scan manipulations required. It is also worth noting that the optimisation discussed in this paper was for an entire socket scan, and further study should be conducted to optimise photo positions for external surface only scans.

Despite the challenges raised in this paper, photogrammetry still offers an inexpensive alternative to conventional optical scanners, with the largest cost being attributed to software—Autodesk ReCap Photo used in this instance. However, freeware alternatives are available. In addition to the base cost for photogrammetry, this study only required patterned tape and a suitable imaging environment.

The low-cost nature of the method proposed in the present study should be weighed against the computation time to generate the mesh scans. Autodesk ReCap utilises cloud computing and, depending on workload, there can be a queue for solving meshes. It was not possible to record the exact solver time but, on average, the process took between half an hour and several hours. When trialling photogrammetry freeware, such as Meshrooms (Alice Vision, France), the solving process took a few hours for each scan, which would vary depending on the computer hardware. The other consideration for Autodesk ReCap is that multiple scans can be solved simultaneously, without compromising solver time, reducing delay for high scan loads. Other authors have noted that there is a large discrepancy in the quality of scans produced from different photogrammetry software packages, which should be investigated prior to use [40].

Further consideration should be made to the continual improvement and development in both the photogrammetry and smartphone industry, meaning that it could become more accessible and relevant as its popularity grows [41].

## 5. Conclusions

This paper presents a low-cost smartphone photogrammetry technique for scanning entire prosthetic sockets. This technique was identified and refined using genetic algorithm optimisation. The evaluation was conducted using a low-cost alternative to the conventional meteorological scanning technique, using digital and physical twins, and 3D printing.

Whilst the optimisation process was considered successful, the model analysis indicated that the specific technique identified for direct photogrammetry of transtibial sockets was not a suitably accurate method for clinical use.

For some applications a modified external socket surface scan would be sufficient to recreate a socket profile, but this incurs increased computational cost and skill requirements.

The optimal gene set simulated led to a socket scan with 38.37% volume difference to the control model across 82 photos. This scan had an average wall thickness of 3.30mm ranging from 2.075 mm to 7.758 mm. In contrast, the socket constructed from the external scan wall had a volume percentage difference of 3.37%, still using 82 photos but with additional computation time required for mesh manipulation.

Further study should be conducted to determine the optimal camera position for external scanning, as well as potentially simpler patterning techniques. A scan of the cast used to form a socket could also be useful for clinical applications and is worth further exploration.

The evaluation of this technique was based on the existing literature for accuracy requirements; clinical trials of the scanned socket would create a better evaluation of socket fit.

## Figures and Tables

**Table 1:** Gene code sample.

Gene Generator																													
A1	G1	A2	G2	A3	G3	A4	G4	A5	G5	A6	G6	A7	G7	A8	G8	A9	G9	A10	G10	A11	G11	A12	G12	A13	G13	A14	G14	A15	G15
45	1	15	1	30	0	30	1	30	0	15	0	30	0	60	1	60	1	15	0	15	0	60	0	45	0	30	1	45	1

**Table 2:** Gene breeding example.

Name	Gene Breeder																													
	A1	G1	A2	G2	A3	G3	A4	G4	A5	G5	A6	G6	A7	G7	A8	G8	A9	G9	A10	G10	A11	G11	A12	G12	A13	G13	A14	G14	A15	G15
**22FBG12**	45	0	60	1	30	0	30	1	60	0	45	1	60	0	60	1	30	0	15	1	45	1	45	1	60	1	45	1	15	0
**22FBG5**	60	1	15	0	15	0	15	0	60	1	30	1	30	1	45	0	45	1	15	1	30	0	15	0	45	1	60	1	45	0
**Child Gene 1**	45	0	60	1	30	0	30	1	60	0	45	1	60	0	60	1	30	0	15	1	30	0	15	0	45	1	60	1	45	0
**Child Gene 2**	60	1	15	0	15	0	15	0	60	1	30	1	30	1	45	0	45	1	15	1	45	1	45	1	60	1	45	1	15	0

**Table 3:** Gene mutation example.

Mutation	Mutator																													
10%	A1	G1	A2	G2	A3	G3	A4	G4	A5	G5	A6	G6	A7	G7	A8	G8	A9	G9	A10	G10	A11	G11	A12	G12	A13	G13	A14	G14	A15	G15
**22FBG3-13**	45	0	60	0	60	0	30	0	30	0	30	0	60	1	60	1	60	0	30	1	15	1	60	0	45	1	15	0	60	1
**M Gene**	45	0	45	0	60	0	30	1	30	0	30	0	60	0	60	1	45	0	45	1	15	1	60	0	60	1	15	0	60	1

**Table 4:** Final camera positions for most fit gene series (22FBG3-14).

Distance d (cm)	Height h (cm)	Angle Increments Ө (°)
150	20	60
100	−10	30
100	50	45
50	20	60
30	20	15
30	50	30
10	50	60
10	80	45
